# Supplementary figures and images for: Morphology of mitochondria in spatially restricted axons revealed by cryo-electron tomography
Source: PLoS Biol. 2018 Sep 17;16(9):e2006169. doi: 10.1371/journal.pbio.2006169 (PMC6160218; doi:10.1371/journal.pbio.2006169)

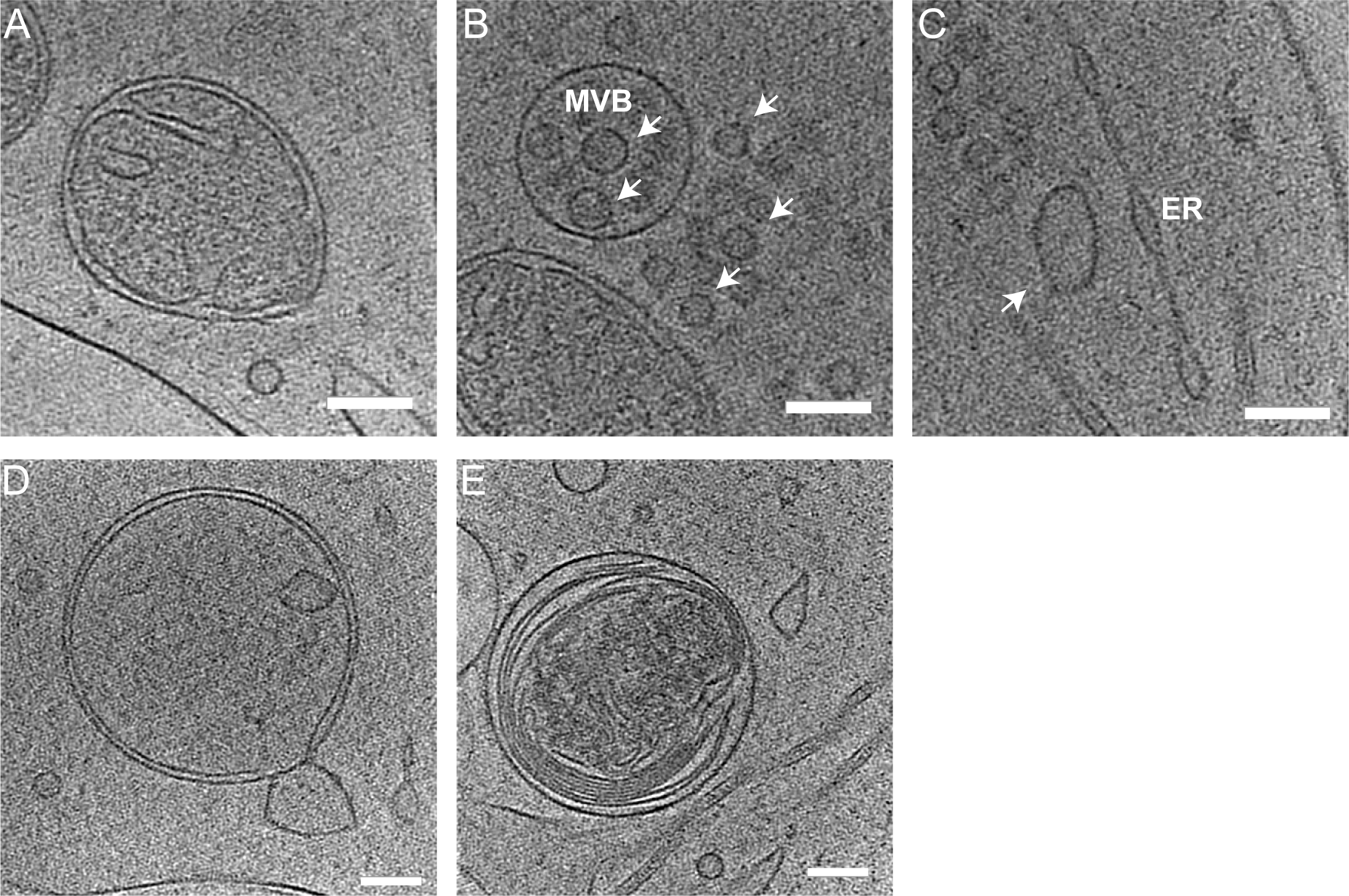

Supplement: S1 Fig — (A) mitochondrion, (B) MVB and vesicles (arrowheads). (C) ER and an unidentified membrane-bound compartment (arrowhead), (D) Autophagosome, (E) Lamellar body. Scale bar = 200 nm. ER, endoplasmic reticulum; MVB, multivesicular body (TIF) [file pbio.2006169.s002.tif]

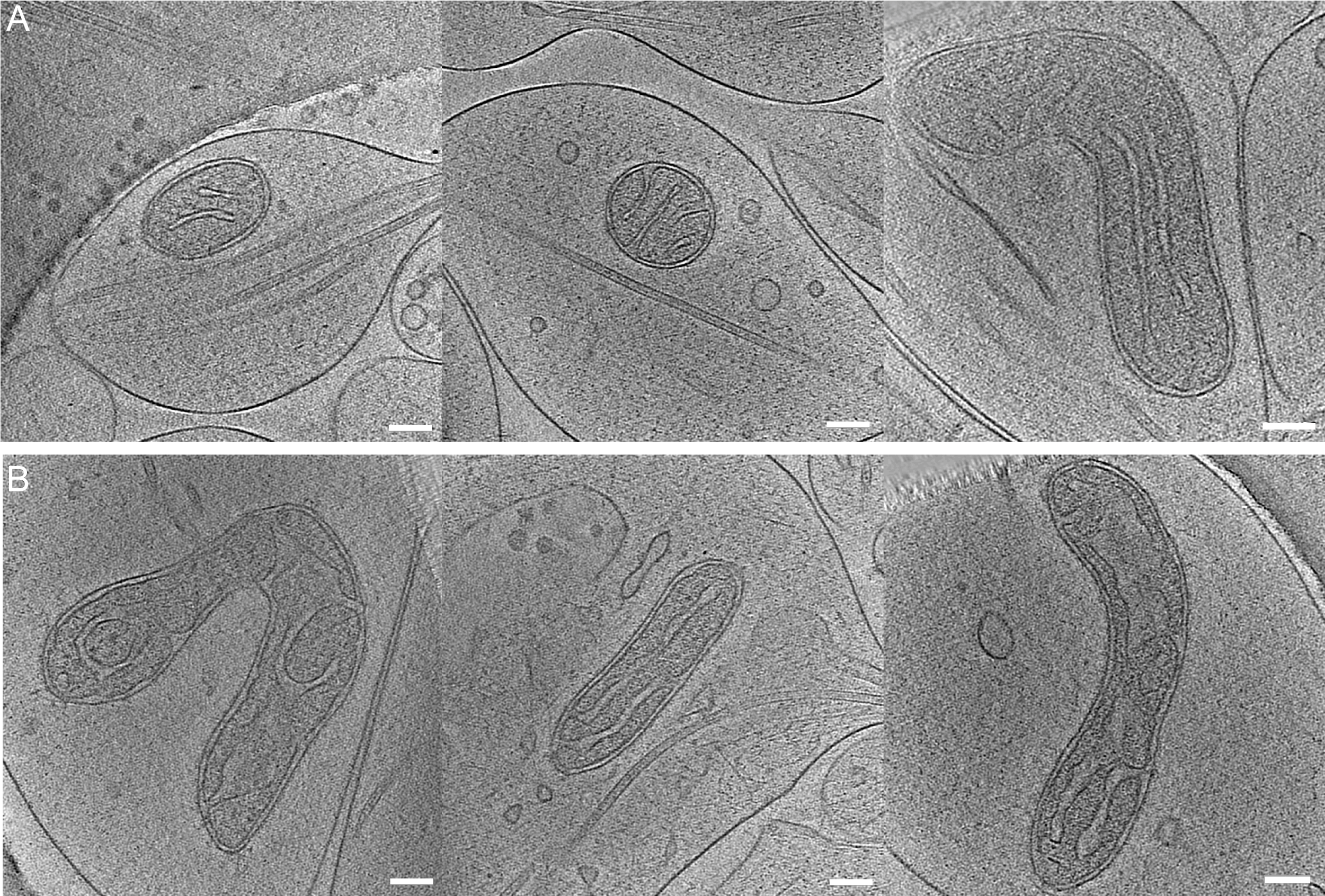

Supplement: S2 Fig — (A) Representations of mitochondria displaying thin, tubulated cristae. (B) Representations of mitochondria displaying thick, unstructured cristae. Populations were identified based on full 3D tomographic reconstructions. Scale bar = 200 nm. (TIF) [file pbio.2006169.s003.tif]
